# CNP attenuates renal toxicity induced by cisplatin in murine kidney

**DOI:** 10.1186/2050-6511-16-S1-A61

**Published:** 2015-09-02

**Authors:** Toru Kimura, Takashi Nojiri, Hiroshi Hosoda, Mikiya Miyazato, Kenji Kangawa

**Affiliations:** 1 Department of Biochemistry, National Cerebral and Cardiovascular Center Research Institute, Suita, Osaka, Japan; 2 Department of General Thoracic Surgery, Osaka University Graduate School of Medicine, Suita, Osaka, Japan

## Background

Cytotoxic chemotherapy is the standard treatment in most cancer patients with advanced stage. However, many patients suffer from severe side effects even if the anti-cancer treatment is effective. There is no established strategy to prevent wide range of side effects of cytotoxic chemotherapy including acute renal injury. Cisplatin is a highly effective chemotherapeutic agent used to treat various malignancies, but its utility is compromised by its nephrotoxicity. C-type natriuretic peptide (CNP), a member of the natriuretic peptide family, exhibits anti-inflammatory effects by activating its specific receptor, guanylyl cyclase (GC)-B. CNP and GC-B receptor are known to be expressed in both the vascular endothelium and kidney. However, there is no evidence about CNP protection against tissue injury by cisplatin, which is a representative cytotoxic agent for most cancer treatment. This study was designed to examine whether CNP pre-treatment attenuates tissue injury including acute renal failure induced by cisplatin.

## Methods

We used C57/B6 mice which were pre-treated with saline or CNP (subcutaneously via osmotic-pump, 2.5μg/kg/min) and administered intraperitoneally a dose of 20 mg/kg cisplatin as the experimental acute renal failure mode. CNP infusion was started one day before the administration of cisplatin. This dose does not change blood pressure and heart rate in mice. At 72 hours after cisplatin injection, urine, blood and kidney samples were collected. Urine and blood samples were examined biochemically. Histological findings and gene expression in kidney tissue were evaluated.

## Results

CNP reduced histological renal tubular damage and apoptosis induced by cisplatin, and suppressed plasma blood urea nitrogen (162 ± 34 vs. 63.0 ± 9.9 mg/dL, p<0.05) and creatinine levels (1.11 ± 0.44 vs. 0.10 ± 0 mg/dl, p<0.05), which were elevated by cisplatin administration. CNP treatment decreased the expression of kidney injury molecule-1 (by 58.6%, p<0.05) and monocyte chemoattractant protein-1 (by 67.6%, p<0.05), which were elevated in the kidney by cisplatin administration. CNP treatment attenuated the decrease in GC-B expression in cisplatin-induced kidney injury.

## Conclusions

The present study is the first to show that CNP inhibits nephrotoxicity and kidney cell damage induced by cisplatin. Our data provide novel insights into the prophylactic therapy for various side effects induced by cytotoxic chemotherapy in many cancer patients.

